# Change in adduction moment following medial open wedge high tibial osteotomy: a meta-analysis

**DOI:** 10.1186/s12891-019-2472-9

**Published:** 2019-03-06

**Authors:** Jun-Ho Kim, Hyun-Jung Kim, Haluk Celik, Joo-Hwan Kim, Dae-Hee Lee

**Affiliations:** 1 Department of Orthopaedic Surgery, Samsung Medical Center, Sungkyunkwan University School of Medicine, Seoul, Korea; 2 Department of Preventive medicine, Korea University College of Medicine, Seoul, South Korea; 3 Department of Orthopaedic Surgery, Umraniye Training and Research Hospital, Istanbul, Turkey

**Keywords:** Adduction moment, Alignment, Knee, Open wedge high tibial osteotomy

## Abstract

**Background:**

This meta-analysis was designed to quantify adduction moment loss, to evaluate the relationship between changes in mechanical axis alignment and adduction moment, and to assess whether sagittal plane moment is altered after medial open wedge high tibial osteotomy (HTO).

**Methods:**

Following preferred reporting items for systematic reviews and meta-analyses (PRISMA) guidelines, all studies reporting preoperative and postoperative peak knee adduction moment or change in peak knee adduction moment from before to after surgery in patients who underwent medial open wedge HTO were included.

**Results:**

Nine studies were included in the meta-analysis. The pooled mean difference in adduction moment from before to after medial open wedge HTO was 1.44% Nm/body weight (BW)xheight (HT) (95% confidence interval [CI]: 1.33 to 1.55% Nm/BWxHT; *P* < 0.001; I^2^ = 4%). However, flexion (0.18% Nm/BWxHT, 95% CI: -0.50 to 0.86% Nm/BWxHT; *P* = 0.61; I^2^ = 79%) and extension (0.15% Nm/BWxHT, 95% CI, − 0.37 to 0.68% Nm/BWxHT; *P* = 0.56; I^2^ = 46%) moments did not differ significantly from before to after surgery. Alignment correction amount and postoperative final valgus alignment were not significantly associated with difference in adduction moment from before to after surgery.

**Conclusion:**

Knee adduction moment after medial open wedge HTO decreased to 60% of the preoperative level. However, this adduction moment decrement was not affected by the magnitude of alignment correction. In addition, there was no change in sagittal plane knee moment, including flexion and extension moments, from before to after medial open wedge HTO.

Level of Evidence: Meta-analysis (Level II).

## Background

Medial open wedge high tibial osteotomy (HTO) is an established procedure for medial compartment osteoarthritis in relatively young active patients [1]. The key to success of HTO is the magnitude of correction, which influences load distribution borne by the knee joint [2]. Load distribution on the knee joint can be determined statically by radiographic measurements of mechanical axis alignment and dynamically by measuring the adduction moment [3]. Adduction moment is defined as the magnitude of the ground reaction force (GRF) passing medial to the knee joint center during gait and the moment arm of the GRF. Knee adduction moment is an indicator of dynamic mechanical load on the knee joint and increases in proportion to knee varus deformity. Although adduction moment has been reported to predict the progression of medial compartment osteoarthritis, there is no general consensus about the magnitude of adduction moment reduction after medial open wedge HTO [4]. Theoretically, the magnitude of the correction in mechanical axis alignment may be closely associated with the change in adduction moment, but previous studies yielded conflicting results regarding the relationship between these two parameters [5, 6]. In addition, although medial open wedge HTO can affect the biomechanical moment in the sagittal plane due to unintended changes in posterior tibial slope, little is known about the change in sagittal plane moment after medial open wedge HTO [7]. This meta-analysis was performed to quantify adduction moment loss, to determine the relationship between change in mechanical axis alignment and change in adduction moment, and to assess whether the sagittal plane moment is altered after medial open wedge HTO. It was hypothesized that the decrement in adduction moment is closely associated with the magnitude of correction in mechanical axis alignment, and that medial open wedge HTO alters the biomechanical environment of the knee joint, not only in adduction moment on the coronal plane but also in flexion and/or extension moments on the sagittal plane.

## Methods

### Literature search

The present meta-analysis was performed in accordance with the guidelines of the preferred reporting items for systematic reviews and meta-analysis (PRISMA) statement and Cochrane Review methods. Comprehensive databases, including EMBASE, PubMed, and the Cochrane Library were sought for studies comparing the pre- and postoperative adduction moment of the knee joint in the patients who underwent open wedge HTO up to May 2018. No restrictions were considered for language or year of publication. Search terms including “Osteotomy” [tiab] OR “Tibial” [tiab] OR “High” [tiab] OR “Open” [tiab], AND “Osteotomy” [MeSH] OR “adduction moment” [tiab] were sought in the title, abstract, MeSH, and keyword fields. Manual searches were additionally done to detect articles potentially missed by the electronic search.

### Study selection

The titles and abstracts of the selected studies were evaluated by two reviewers, independently. If enough data were not obtained using the abstract, the full text of the article was reviewed. Studies were included according to following conditions: (1) preoperative and postoperative peak knee adduction moment or change in the peak knee adduction moment between prior to and after surgery in the patients who underwent medial open wedge HTO were reported; (2) primary outcomes included change in the peak knee adduction moment, standardized to body weight (BW) and height (HT); and (3) the numbers of samples included in the final analysis with means and standard deviations of adduction moment. Studies were excluded if (1) they reported only either preoperative or postoperative adduction moment; or (2) they did not include appropriate information, such as standard deviation or range of values.

### Data extraction

Data was extracted from each study by two authors using a data extraction form. A third author reviewed the data in case of any disagreement.

The variables were noted as following: (1) mean and standard deviations of the peak adduction moments of the knee joint and mechanical axis alignment before and after medial open wedge HTO; (2) sample size; and (3) study type (e.g. prospective or retrospective comparison studies). In case of the absence of the variables, the corresponding author of the related study was asked to provide the necessary data via e-mail. The corresponding authors of two studies responded our requests and shared the necessary information.

### Assessment of methodological quality

The Newcastle-Ottawa Scale was used to define the methodological qualities of each study by two reviewers, independently [8], as recommended by the Cochrane Non-Randomized Studies Methods Working Group. The Newcastle-Ottawa Scale includes the following criteria: the comparability of the groups, the definition of the study groups and the cohort studies identification of either the exposure or outcome of interest for case–control or. Studies had scores > 5 points determined as a high quality study. Disagreements related to the scores between reviewers were solved by discussion and consensus.

### Statistical analysis

Mean differences in the peak knee adduction moments prior to and after medial open wedge HTO were the main outcomes of the meta-analysis. Random-effects meta-analyses were implemented to collect these outcomes through included studies, by estimating the weighted mean differences, and 95% confidence intervals (CIs). To define the heterogeneity among studies, I^2^ statistics was used by estimating the amount of irregularities due to actual differences between studies, rather than differences due to random error or chance. The values of 25, 50, and 75% were considered as low, moderate, and high, respectively.

Statistical analysis was performed using RevMan version 5.2 (Copenhagen, the Nordic Cochrane Centre, The Cochrane Collaboration, 2012). The risks of bias were independently assessed by two investigators and were indicated as either low, high or unclear.

A random-effects meta-regression was used by assuming that the true effects follow a normal distribution around the linear predictor.

A meta-regression analysis was performed to evaluate the influence of alignment correction amount and postoperative alignment on change of adduction moment from before to after high tibial osteotomy. The permutation test based on Monte Carlo simulation was conducted to adjust the risk of false-positive findings with multiple covariates using few studies.

## Results

### Identification of studies

**Fig. 1:**
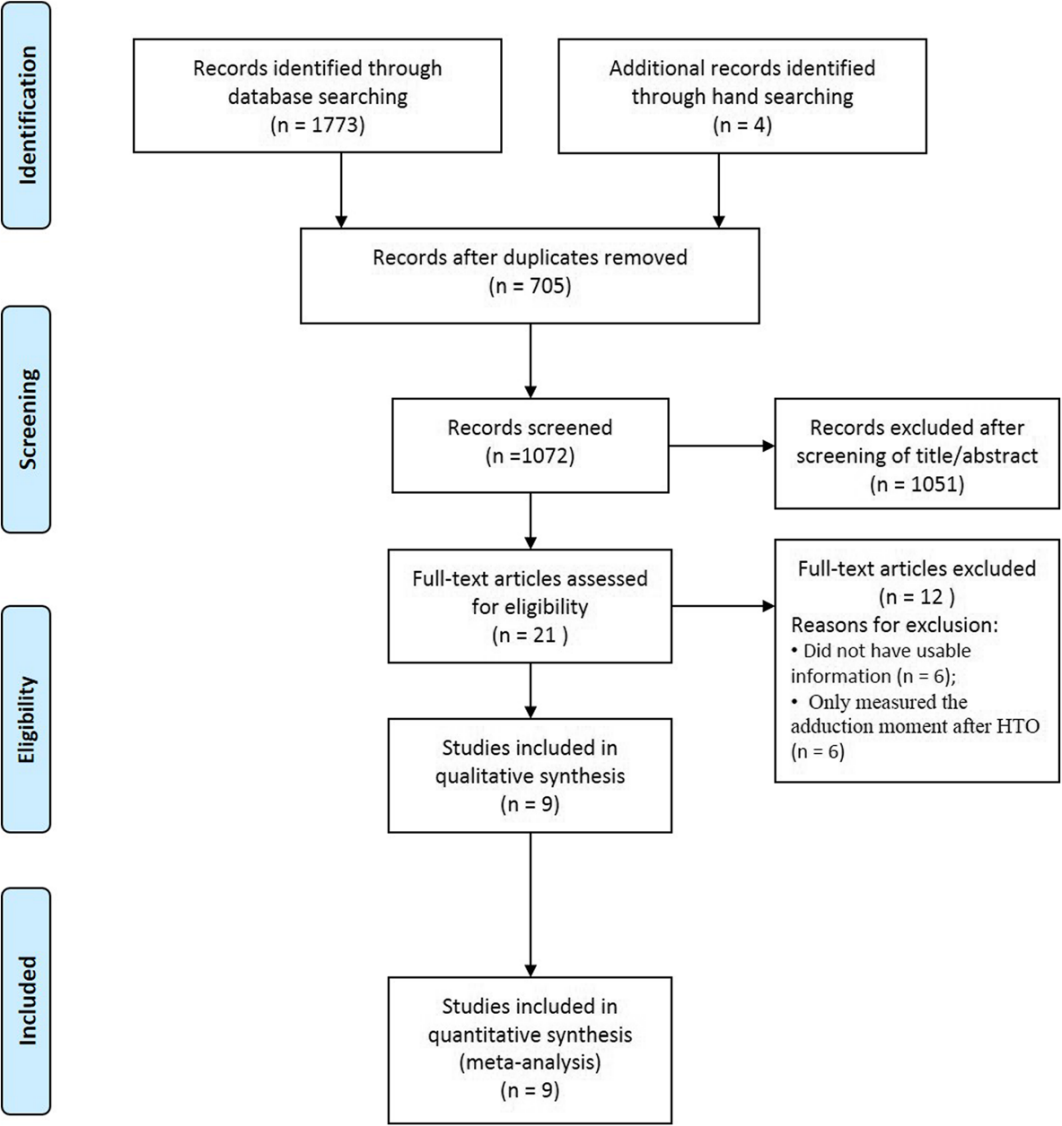
PRISMA (Preferred Reporting Items for Systematic reviews and Meta-analyses) flow diagram of the identification and selection of the studies included in this meta-analysis

Figure 1 presents the details of study identification, inclusion, and exclusion. An initial electronic search yielded 358 studies in PubMed (MEDLINE), 509 in EMBASE, 382 in Web of Science, 503 in SCOPUS, and 22 in the Cochrane Library. In additon, four studies were identified through manual searching. Once 705 duplicates were eliminated, 1051 of the remaining 1072 studies were excluded based on reading of the abstracts and full-text articles. An additional 12 studies were excluded due to the absence of the usable information or usage of only either preoperative or postoperative peak adduction moment measuraments. Consequently, nine studies [5, 6, 9–15] were included in the present meta-analysis.

### Study types and methodological quality of the included studies

The final nine studies compared the adduction moment in subjects prior to and after medial open wedge HTO. Flexion and/or extension moments were also compared prior to and after the surgery in five of those. All included studies presented low risk of selection bias. Sufficient follow-up duration was defined as the time interval between surgery and performance of gait analysis, with a shorter time interval associated with a higher risk of bias, due to accurate measurement of adduction moment being unlikely. All included studies included the patients with medial compartment knee osteoarthritis who underwent open HTO with fixation of locking plate. The patient demographic such as age and gender ratio were also similar between studies. These findings could suggest the lower risk of bias for representativeness of cases in each study. Four of eight studies had a high quality level (> 5 points) according to the Newcastle-Ottawa Scale (Table 1).

**Table 1:** Characteristics and quality of studies included in the meta-analysis

Author	Year	Study Type	Sample Size, n	Male/Female Sex, n	Mean Age, y	Measured Parameters	Time from surgery to measurement, mo	Follow-up, Mean, mo	Mechanical axis angle, degrees		Quality score
									Baseline Mean (SD)	Final assessment Mean (SD)	
Birmingham et al. [9]	2009	PCS	126	102	47.48	ADM	24	24	−8.00 (4.09)	−0.05 (3.05)	5
DeMeo et al. [10]	2010	PCS	20	14	49.4	ADM	7.2	99.6	−3.6 (N/R)	7.5 (N/R)	6
Kean et al. [11]	2009	RCS	21	N/R	38.9	ADM, FLM, EXM	12	12	−6.03 (3.29)	0.33 (N/R)	8
Leitch et al. [12]	2015	RCS	14	12	48	ADM, FLM, EXM	12	12	−7.61 (N/R)	0.52 (N/R)	6
Lind et al. [5]	2013	RCS	11	11	46	ADM, FLM, EXM	12	12	−8 (2.8)	0 (2.1)	7
Marriot et al. [13]	2015	PCS	33	N/R	40	ADM, FLM, EXM	60	68	−5.90 (2.87)	1.69 (2.37)	4
McClelland et al. [6]	2016	RCS	36	33	54.11	ADM	72	72	−8.6 (0.88)	−1.44 (1.05)	6
Sischek et al. [14]	2014	RCS	37	29	49.3	ADM	6 48	N/R	−8.35 (3.33)	1.25 (2.67)	8
Birmingham et al. [15]	2017	PCS	170	135	46.4	ADM, FLM	60	60	−8.01 (3.38)	0.28 (2.86)	7

*PCS* prospective comparison study, *RCS* retrospective comparison study, *ADM* adduction moment, *FLM* flexion moment, *EXM* extension moment, *N/R* not reported; For mechanical axis angle, negative values indicate varus alignment

### Adduction moment change after medial open wedge HTO

**Fig. 2:**
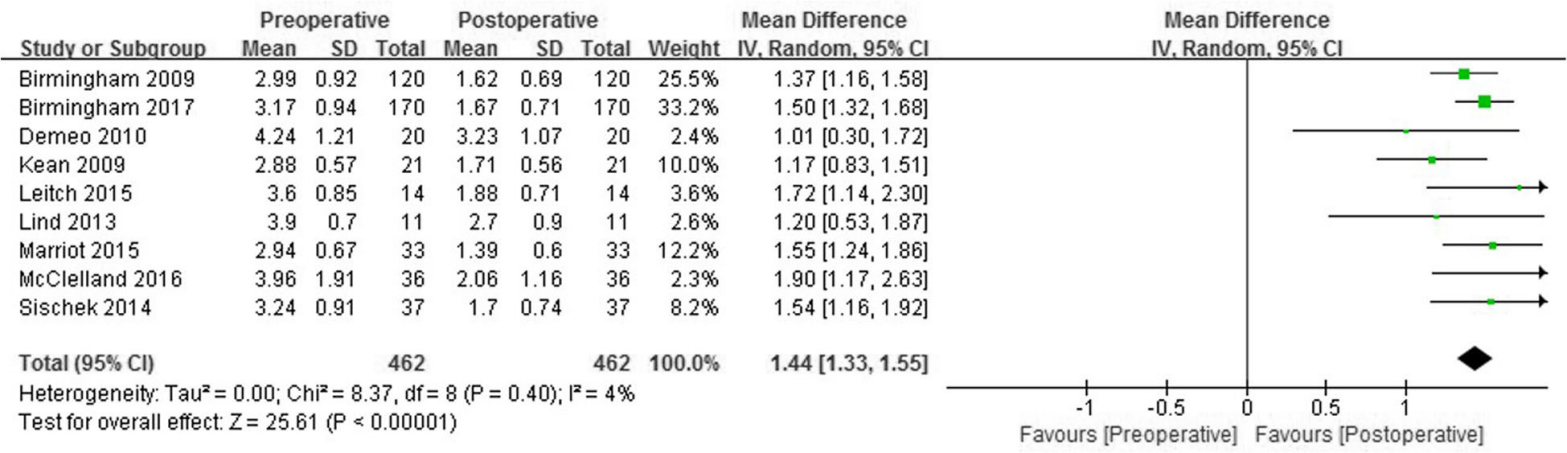
Forest plots of studies showing the change of adduction moment in patients who underwent medial open wedge high tibial osteotomy

A total of 462 knees that underwent medial open wedge HTO were evaluated. The pooled mean difference in adduction moment prior to and after medial open wedge HTO was 1.44% Nm/BWxHT (95% CI: 1.33 to 1.55% Nm/BWxHT; *P* < 0.001; I^2^ = 4%) (Fig. 2). The adduction moment was reduced by roughly 40% of preoperative levels (mean preoperative adduction moment 3.46% Nm/BWxHT).

### Changes in flexion and extension moment after medial open wedge HTO

**Fig. 3:**
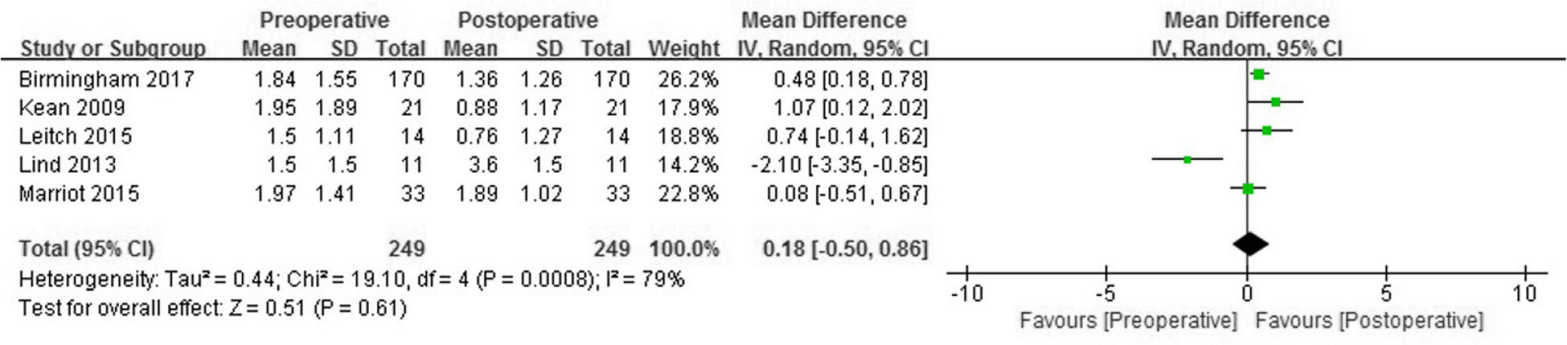
Forest plots of studies showing the change of flexion moment in patients who underwent medial open wedge high tibial osteotomy

**Fig. 4:**
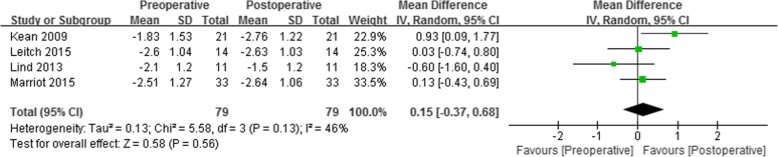
Forest plots of studies showing the change of extension moment in patients who underwent medial open wedge high tibial osteotomy

Of the eight studies, four reported changes of flexion and extension moments in the sagittal plane as well as changes in adduction moment in the coronal plane. Slight mean differences in flexion (0.18 Nm/BWxHT, 95% CI: -0.50 to 0.86% Nm/BWxHT; *P* = 0.61; I^2^ = 79%, Fig. 3) and in extension (0.15 Nm/BWxHT, 95% CI: -0.37 to 0.68% Nm/BWxHT; *P* = 0.56; I^2^ = 46%, Fig. 4) moments were observed, but neither was statistically significant.

### Meta-regression analyses

The results of meta-regression analyses are reported in Table 2. Alignment correction amount and postoperative final valgus alignment were not significantly associated with difference in adduction moment from before to after surgery. This finding indicated that the change in adduction moment from before to after medial open wedge HTO was not influenced by the magnitude of alignment correction or postoperative final valgus alignment. Monte Carlo permutation test was also used to confirm the false positive findings due to small sample size of our including studies. The results of this test also showed no significant association between age (*P* = 0.466) and follow up (*P* = 0.149) and adduction moment change from before to after medial open wedge HTO.

**Table 2:** Meta-regression analyses between correction amount and postoperative valgus, and change in adduction moment from before to after medial open wedge high tibial osteotomy (HTO)

Variable	Coefficient	Standard error	*P* value	95% confidence interval
Change in adduction moment from before to after surgery				
Correction amount	−0.180	0.096	0.159	−0.487 to 0.127
Postoperative valgus	−0.012	0.037	0.767	−0.106 to 0.130

## Discussion

This meta-analysis verified that knee adduction moment decreased markedly after medial open wedge HTO, reaching about 60% of the preoperative level, whereas knee flexion and extension moments were not altered by surgery.

The mechanical force loaded onto the knee joint in patients with medial osteoarthritis and varus deformity is an important predictor of osteoarthritis progression. Although the mechanisms by which increased mechanical loading adversely affects cartilage are unclear, they may involve chondrocyte death, disruption of the extracellular matrix, and microfracture within the subchondral cortical endplate. Knee adduction moment is a surrogate measure of dynamic mechanical load on the knee joint and increases in proportion to knee varus deformity. The success of medial open wedge HTO depends not only on alignment correction but also on decreasing the adduction moment, thereby reducing the mechanical load on the medial compartment of the knee. Determining the magnitude of adduction moment loss and its clinical relevance after medial open wedge HTO is therefore important. This meta-analysis showed that medial open wedge HTO reduced preoperative adduction moment by about 40%. One recent study reported that a 1% increase in adduction moment increased the risk of progression of knee osteoarthritis 6-fold. These findings suggest that a 40% reduction in adduction moment would delay the progression of medial osteoarthritis. Another recent study found that the load on the medial compartment while walking increases 5% for every 1° change towards varus [16]. Our study found that the magnitude of alignment correction was not significantly related to the reduction in adduction moment. Nevertheless, because the mean amount of correction following medial open wedge HTO in the studies included in this meta-analysis was approximately 8°, the 40% reduction in adduction moment compared with the preoperative level may be explained by the results showing a 5% decrease in adduction moment per 1° change towards varus.

The knee adduction moment is a consequence of the magnitude of the ground reaction force (GRF) passing medial side of the center of the knee joint during gait and the moment arm of the GRF, and defined as the perpendicular distance from the GRF to the center of the knee joint [17]. Therefore, the mechanical axis and knee adduction moment are positively correlated with each other [18]. In contradiction to that knowledge, the results of our meta-analysis revealed an absence of association between the magnitude of alignment correction and/or the degree of valgus and the reduction in adduction moment following medial open wedge HTO. Several causes are possible. First, despite the large amount of the correction, the mechanical axis may not have reached to the ultimate end-point, which was 62.5% of the width of the entire tibial plateau. Second, the adduction moment may have been affected by other factors except the mechanical axis alignment. For instance, in spite of the fact that the mechanical axis was shown as an indicant of the peak adduction moment, it only accounted for roughly 50% of the variation in the peak adduction moment [19]. The foot progression angle, lateral trunk tilt, knee flexion angle, and walking speed may also affect knee adduction moment. Increased lateral trunk tilt and out-toeing gait may decrease the knee adduction moment, whereas bigger knee flexion angle and higher walking speed have been reported as factors that may increase the knee adduction moment [18, 20, 21]. In addition, the strength of extensor and flexor muscles of the knee and the knee joint laxity affect the adduction moment [22–24].

Medial open wedge HTO can affect the sagittal plane moments of the knee during gait. Sagittal plane moments may alter the mechanical load borne by the medial compartment of the knee joint. Previous studies showed conflicting results for change in flexion moment. One study showed increased flexion moment after HTO due to increasing gait speed and flexion angle, and improved quadriceps function resulting from reduced pain following HTO [5]. In contrast, another study found that not only peak adduction moment but also flexion moment were reduced 12 months after medial open wedge HTO [12]. Our meta-analysis showed no significant change in either flexion or extension moment. Differences between study results may be due to the small effect on the sagittal plane of the magnitude of correction of mechanical axis alignment, despite the change in mechanical axis alignment being closely associated with change in adduction moment after medial open wedge HTO. Neuromuscular changes, which widely vary among individuals and are unpredictable, may contribute to changes in sagittal plane moments [24]. An unintended posterior slope of the articular surface of the proximal tibia in the sagittal plane may also affect changes in sagittal plane moments. However, a recent meta-analysis showed that the magnitude of increase in the posterior tibial slope following medial open wedge HTO was only 2°, suggesting that this slight change may have little effect on the biomechanical environment of the knee joint [25]. The results of that study may explain, at least in part, the lack of significant change in sagittal plane moments observed in our study.

This study had several limitations. Although adduction moment is a proxy measure of the mediolateral distribution of loads across the knee, it is not a direct measure of the actual force borne by the knee joint. The angular impulse of knee adduction moment was shown to be a more comprehensive indicator of cartilage volume reduction in osteoarthritis than peak knee adduction moment [4], because the former accounts not only for load magnitude but also load duration, which contribute to total knee load exposure [26]. However, angular impulse of adduction moment has only recently been measured, making peak knee adduction moment a more practical measure, as well as being a valid and reliable dynamic indicator of compressive load in the medial compartment during walking and a useful parameter for comparing the results of studies that assess adduction moment. Another limitation was the variation in preoperative mechanical axis and lateral thrust among included studies, which may have resulted in large variations in data, including adduction moment, during gait. This finding may also be a potential cause of heterogeneity in this meta-analysis [22]. Finally, a meta-regression analysis, which performed in this study to investigate the influence of alignment correction amount and postoperative alignment on change of adduction moment, was also prone to instability in the results due to relatively small sample size.

## Conclusions

Knee adduction moment after medial open wedge HTO decreased to 60% of its preoperative level. However, this adduction moment decrement was not affected by the magnitude of alignment correction. In addition, there was no change in sagittal plane knee moment, including flexion and extension moments, from before to after medial open wedge HTO.

## Abbreviations

BW: Body weight
CI: Confidence interval
HT: Height
HTO: High tibial osteotomy
PRISMA: Preferred reporting items for systematic reviews and meta-analysis
